# A novel *ZIC3* gene mutation identified in patients with heterotaxy and congenital heart disease

**DOI:** 10.1038/s41598-018-30204-3

**Published:** 2018-08-17

**Authors:** Shuolin Li, Sida Liu, Weicheng Chen, Yuan Yuan, Ruoyi Gu, Yangliu Song, Jian Li, Yinyin Cao, Yixiang Lin, Jun Xu, Huijun Wang, Duan Ma, Xiaojing Ma, Wei Sheng, Guoying Huang

**Affiliations:** 1 Children’s Hospital of Fudan University, Shanghai, 201102 China; 2 Shanghai Key Laboratory of Birth Defects, Shanghai, 201102 China; 3 Key Laboratory of Molecular Medicine, Ministry of Education, Shanghai Medical College, Fudan University, Shanghai, 200000 China

## Abstract

Heterotaxy syndrome (HTX) is characterized by left-right (LR) asymmetry disturbances associated with severe heart malformations. However, the exact genetic cause of HTX pathogenesis remains unclear. The aim of this study was to investigate the pathogenic mechanism underlying heterotaxy syndrome. Targeted next-generation sequencing (NGS) was performed for twenty-two candidate genes correlated with LR axis development in sixty-six HTX patients from unrelated families. Variants were filtered from databases and predicted *in silico* using prediction programs. A total of twenty-one potential disease-causing variants were identified in seven genes. Next, we used Sanger sequencing to confirm the identified variants in the family pedigree and found a novel hemizygous mutation (c.890G > T, p.C297F) in the *ZIC3* gene in a male patient that was inherited from his mother, who was a carrier. The results of functional indicated that this *ZIC3* mutation decreases transcriptional activity, affects the affinity of the GLI-binding site and results in aberrant cellular localization in transfected cells. Moreover, morpholino-knockdown experiments in zebrafish demonstrated that *zic3* mutant mRNA failed to rescue the abnormal phenotype, suggesting a role for the novel *ZIC3* mutation in heterotaxy syndrome.

## Introduction

Heterotaxy (HTX) is a rare multiple congenital anomaly syndrome resulting from abnormal specification of the left-right (L-R) body axis or ciliary dysfunction during embryonic development^1–3^. HTX is characterized by a wide variety of cardiac or extracardiac congenital malformations, causing significant mortality or morbidity^4^. The incidence of HTX is 1 per 10,000–20,000 live births, and over 90% of HTX patients present with complex cardiovascular malformations^4^.

However, the exact genetic cause of HTX remains unclear. Although HTX typically occurs sporadically, the associated relative risk is highest for cardiovascular malformations, indicating the existence of a strong genetic component^5^. Studies have identified autosomal recessive, autosomal dominant and X-linked inheritance patterns in HTX patients, accompanied by congenital heart defects (CHDs)^5,6^. A series of loci and disease genes related to HTX have also been identified^5^, and point mutations in more than 15 genes, such as *ZIC3*, have been identified in humans with HTX or HTX-spectrum CHD^7^. The *ZIC3* (*zinc-finger in cerebellum 3*; GenBank: AF028706) gene is located at Xq26.2, which is the location of the genetic cause of X-linked heterotaxy (MIM #306955) and regulates early embryonic patterning in vertebrates^8^. The *ZIC3* gene is a Cys2His2 (C2H2) zinc-finger transcription factor belonging to the GLI super family involved in LR axis development and binding to the GLI consensus binding site (GLIBS). Mutations in the *ZIC3* gene accounting for approximately 1% of sporadic and 75% of X-linked familial heterotaxy have been identified, which have also been associated with cases of isolated d-transposition of the great arteries (TGAs) and double-outlet right ventricle (DORV), suggesting that this gene is highly correlated with LR asymmetry^9–12^. Studies utilizing mouse and *Xenopus* models have demonstrated the critical role of *ZIC3* expression during both embryonic gastrulation and cardiac development^13,14^.

Previous studies have identified a panel of twenty-two genes related to LR asymmetry and cilia function that are involved in HTX syndrome: *LEFTY1*, *LEFTY2*, *CFC1*, *ACVR2B*, *TGFBR2*, *RPSA*, *CRELD1*, *SHROOM3*, *GJA1*, *FOXH1*, *INVS*, *NODAL*, *NAT10*, *BCL9L*, *NEK8*, *MEGF8*, *SMAD2*, *ZIC3*, *DNAH5*, *ARMC4*, *CFAP53*, and *NPHP4* (details provided in Supplementary Table S1)^7,12,15–27^.

Although there is a strong genetic contribution to laterality defects, most cases remain unexplained, indicating that the utilization of novel genomic approaches to identify genetic causes of these complicated disease is necessary. Given the mutational spectrum of heterotaxy, we hypothesized that NGS approaches could help identify novel and essential variants to improve our understanding the contribution of susceptibility alleles to disease^7,28^. Our study provides important clues for understanding the pathogenic mechanism of *ZIC3* gene mutations in heterotaxy syndrome and expands the available data on the spectrum of gene mutations associated with the etiology of this disease.

## Results

### The *ZIC3* mutation identified in the heterotaxy pedigree

We performed next-generation sequencing (NGS) in a cohort of sixty-six patients with HTX and congenital heart disease (CHD) from unrelated families. Patient clinical characteristics are summarized in Table 1. Sequencing was focused on the exome of the following twenty-two candidate genes correlated with LR axis development: *NPHP4*, *LEFTY1*, *LEFTY2*, *CFC1*, *ACVR2B*, *TGFBR2*, *RPSA*, *CRELD1*, *SHROOM3*, *DNAH5*, *GJA1*, *FOXH1*, *INVS*, *ARMC4*, *NODAL*, *NET43*, *BCL9L*, *NEK8*, *CCDC11*, *MEGF8*, *SMAD2* and *ZIC3*. These genes were interrogated for novel or rare coding variants present only in heterotaxy patients with CHD. By comparing with dbSNP, 1000 Genome Project, and NHLBI Exome Sequencing Project databases and using prediction programs, we excluded common variants, synonymous mutations and non-synonymous mutations that are predicted to have no deleterious effect on protein function. Twenty-one potential disease-causing variants were identified in seven genes: *DNAH5*, *ARMC4*, *MEGF8*, *SHROOM3*, *NPHP4*, *ACVR2B* and *ZIC3*. Eight of these variants were novel, and thirteen were low-frequency variants (MAF <1% SNP) (Table 2).

**Table 1:** Demographics and clinical characteristics of the patients (n = 66).

Variable	N or mean	% or range
Gender		
Male	46	70
Female	20	30
Age (month) [Median (IQR)]	36 (9.5–78)	
Visceral situs		
Solitus	0	0
Inversus	13	20
Ambiguous	53	80
Cardiac position		
Levocardia	24	36
Dextrocardia	40	60
Mesocardia	2	3
Combined cardia phenotype		
ASD	28	42
VSD	23	35
AVSD	20	30
TGA	34	52
DORV	22	33
DOLV	4	6
BSVC	10	15
PLSVC	5	8
TAPVD	5	8
Single atrium	8	12
Atrium isomerism	15	23
Ventricular morphology		
Two ventricles of equal size	41	62
Single Ventricle	25	38
Ventricular loop (%)		
D-loop	28	42
L-Loop	31	47
Unknown	7	11
Spleen morphology, position		
Normal	35	53
Asplenia	16	24
Polyspenia	3	5
Right side spleen	12	18
Liver		
Normal	40	61
Reverse	13	20
Middle	13	20
Stomach		
Normal	53	80
Reverse	12	18
Middle	1	2

Detailed cardiac anatomy of children with HTX. IQR, Interquartile range; ASD, atrial septal defect; VSD, ventricular septal defect; AVSD, atrioventricular septal defect; TGA, transposition of the great arteries; DORV, double outlet right ventricle; DOLV, double outlet left ventricle; BSVC, bilateral superior vena cava; PLSVC, persistent left superior vena cava; TPAVD, total anomalous pulmonary venous drainage.

**Table 2:** All novel and rare genetic variants detect in the 22 genes.

Gene	Mutated case	CDS	Amino Acid change	zygosity	rsID	MAF	SIFT	Polyphen2	MutationTaster	ExAc
DNAH5 (NM_001369)	5032	c.13364C > A	p. Gly4455Asp	Het	rs370684795	0.0002	D	D	D	0.0003135
	5041	c.12367C > T	p. His4123Tyr	Het	rs151145750	0.0008	D	P	D	0.0007023
	5056	c.12595C > T	p. Arg4199Cys	Het	rs374874272	0.00002	D	D	D	0
	5063	c.10169A > G	p. Asp3390Gly	Het	This study	NA	D	B	D	0
	5042	c.7123A > T	p. Ile2375Phe	Het	rs529696058	0.00002	D	P	D	0.0003469
	5078/5133	c.6053T > C	p. Ile2018Thr	Het	rs117989731	0.0004	D	D	D	0.001041
	5102/5145/5188	c.12472C > T	p. Arg4158Trp	Het	rs3756672	0.0130	D	D	D	0.004219
	5119	c.9781A > G	p. Lys3261Glu	Het	rs146215039	0.0006	T	B	D	0.0002636
	5138	c.9236G > A	p. Arg3079Gln	Het	This study	NA	T	P	D	0.00008246
ARMC4 (NM_018076)	5071	c. 1679C > T	p. Ala560Val	Het	This study	NA	D	D	D	0.00002482
MEGF8 (NM_001410)	5071	c.3109C > T	p. Arg1037Trp	Het	rs370522595	0.0005	D	D	D	0.0004647
	5041	c.8068C > A	p. Pro2690Thr	Het	This study	NA	T	P	D	0
SHROOM3 (NM_020859)	5052	c.580C > A	p. His194Asn	Het	This study	NA	D	D	D	0
	5101/5126	c.4726A > G	p. Lys1576Glu	Het	rs1396351	0.0016	D	P	N	0.0008678
	5138	c.2905C > T	p. Arg969Trp	Het	rs3733245	0.00842	D	B	D	0.001332
NPHP4 (NM_015102)	5056	c.2198G > A	p. Gly733Asp	Het	rs587783027	0.0001	D	D	D	0.001476
	5050	c.880G > A	p. Gly294Ser	Het	This study	NA	D	D	D	0
	5020/5072	c.694C > T	p. Arg232Cys	Het	rs572497035	0.0001	D	D	D	0.0001258
	5044	c.3160C > T	p. Arg1054Cys	Het	rs373369949	0.0002	D	D	D	0.002275
ACVR2B (NM_001106)	5204	c.1219G > A	p. Val407Met	Het	This study	NA	D	P	D	0
ZIC3 (NM_003413)	5183	c.890G > T	p. Cys297Phe	Hemi	This study	NA	D	D	D	0

MAF, minor allele frequency; the date is from NCBI dbSNP data base and we use 1000 Genomes data.

Exome Aggregation Consortium (ExAC) version 3: minor allele frequencies for individuals of European descent are shown.

T: tolerant; P: probably damage; D: disease causing; B: Benign; Het: Heterozygous; Hemi: Hemizygous.

**Figure 1:**
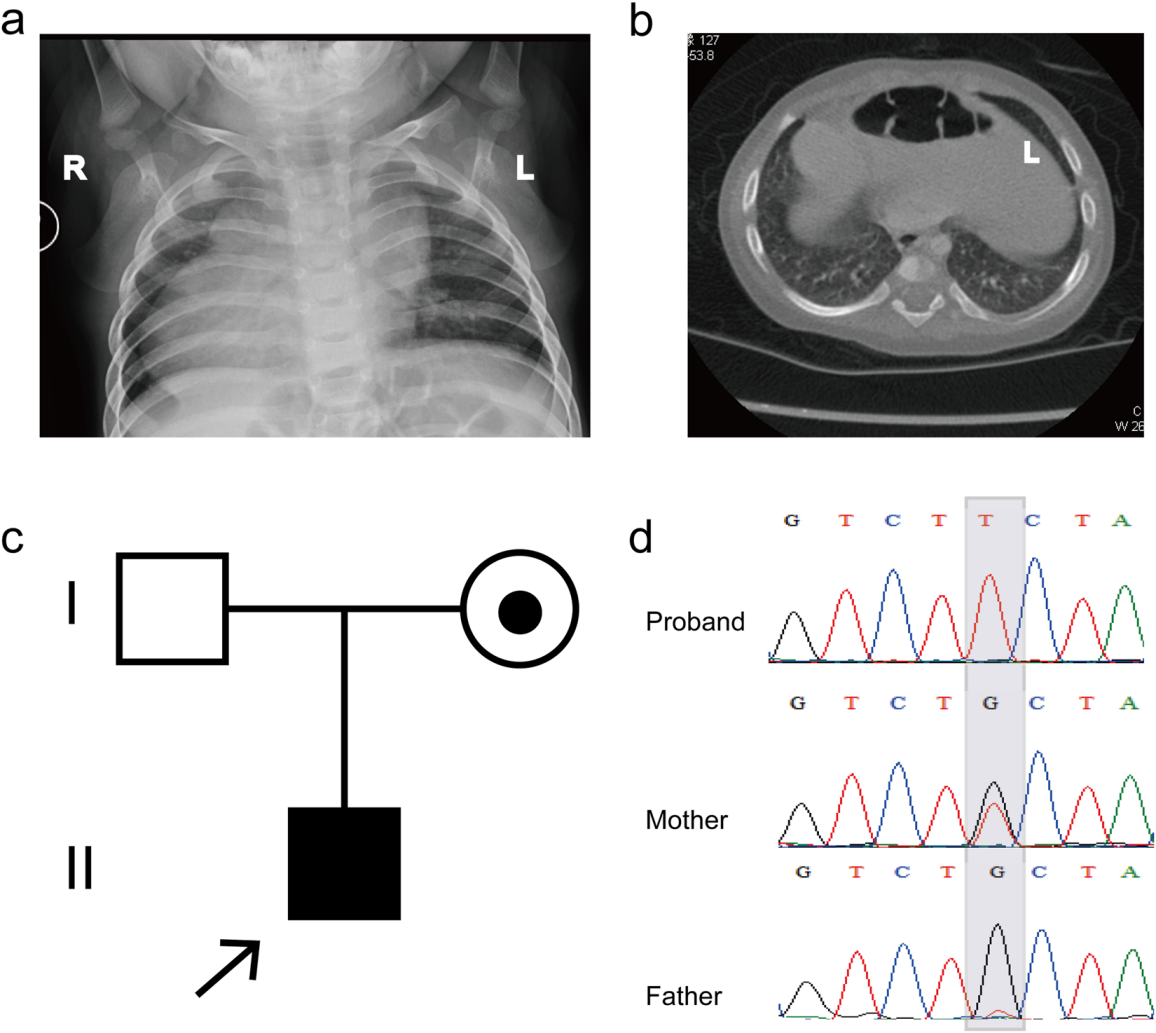
Clinical features of the proband and the *ZIC3* gene mutation. (**a**) Chest radiograph showing dextrocardia in the patient. (**b**) CT scan indicating that the liver and spleen of the patient are reversed, while the stomach is located in the middle of the body. (**c**) The trio family of the proband, in which the mother is a carrier. (**d**) Sanger sequencing results show that the hemizygous *ZIC3* mutation was found in the proband, while his father did not carry this mutation, but his mother was a heterozygous carrier. The gray background indicates the nucleotide change from G to T in the patient at position 890.

We used Sanger sequencing to confirm the variants observed in the family pedigrees and identified a novel hemizygous mutation (c.890G > T, p.C297F) in the X-linked *ZIC3* gene in a 13-month-old male patient with asplenia syndrome (also known as right atrial isomerism), right stomach, left liver, TGA and DORV (Fig. 1a and b). This novel hemizygous mutation was inherited from the proband’s carrier mother and was not observed in his father (Fig. 1c and d). We then confirmed the *ZIC3* mutation (c.890G > T, p.C297F) via Sanger sequencing in 200 healthy control and 100 sporadic HTX cases but did not detect the mutation in any of the samples.

**Figure 2:**
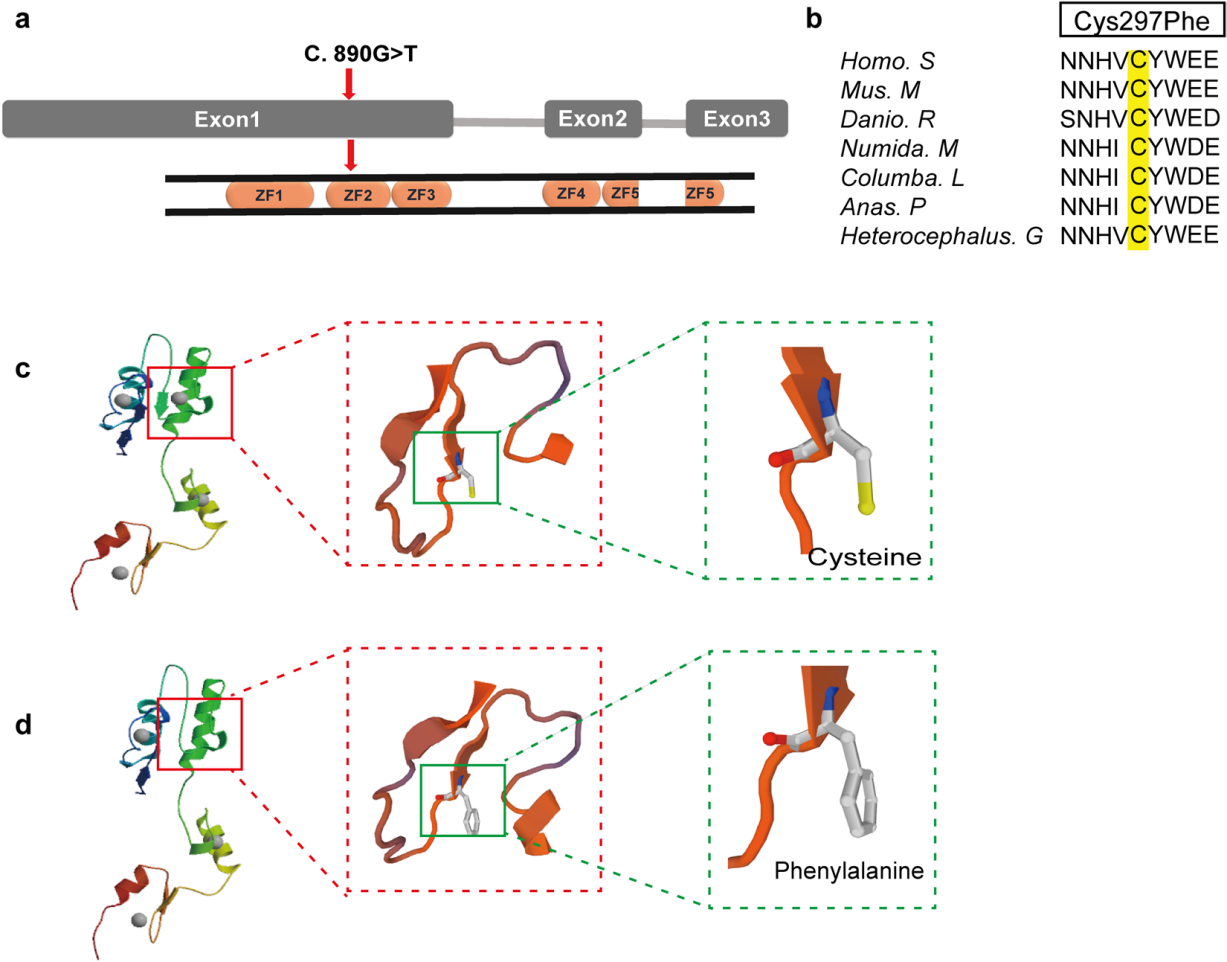
Position of the *ZIC3* variant and the corresponding protein structure change. (**a**) The position of the mutation at the end of exon one, corresponding to the third amino acid of the second C2H2 domain of the ZIC3 protein. (**b**) Alignment of multiple ZIC3 protein sequences among several species. The altered amino acid boxed in yellow is completely conserved evolutionarily across various species. Homo. S: *Homo sapiens*; Mus. M: *Mus musculus*; Danio. R: *Danio rerio*; Numida. M: *Numida meleagris*; Columba. L: *Columba livia*; Anas. P: *Anas platyrhynchos*; Heterocephalus. G: *Heterocephalus glaber*. (**c**,**d**) Comparison of the wild-type ZIC3 protein structure with the mutant using SWISS-MODEL. Panel c shows that wild-type ZIC3 harbors a cysteine at amino acid No. 297, and the ZIC3 protein structure contains four ligands. Panel d indicates that variant changed the amino acid residue from cysteine to phenylalanine, indicating that only three ligands exist in the mutant protein.

The identified *ZIC3* mutation (c.890G > T, p.C297F) was located in exon one, corresponding to the third amino acid of the second C2H2 domain of the ZIC3 protein (Fig. 2a). The p.Cys297 residue is conserved from humans to *Heterocephalus* (based on Ensembl browser genomic alignments) (Fig. 2b), suggesting that this amino acid is crucial for protein function. Using SWISS-MODEL, we compared the 3D structure of the wild-type ZIC3 protein and the mutant protein (p.C297F), which indicated that the mutant prevents the binding by ZIC3 (Fig. 2c).

### Effect of the *ZIC3* mutation on DNA binding and gene transactivation

**Figure 3:**
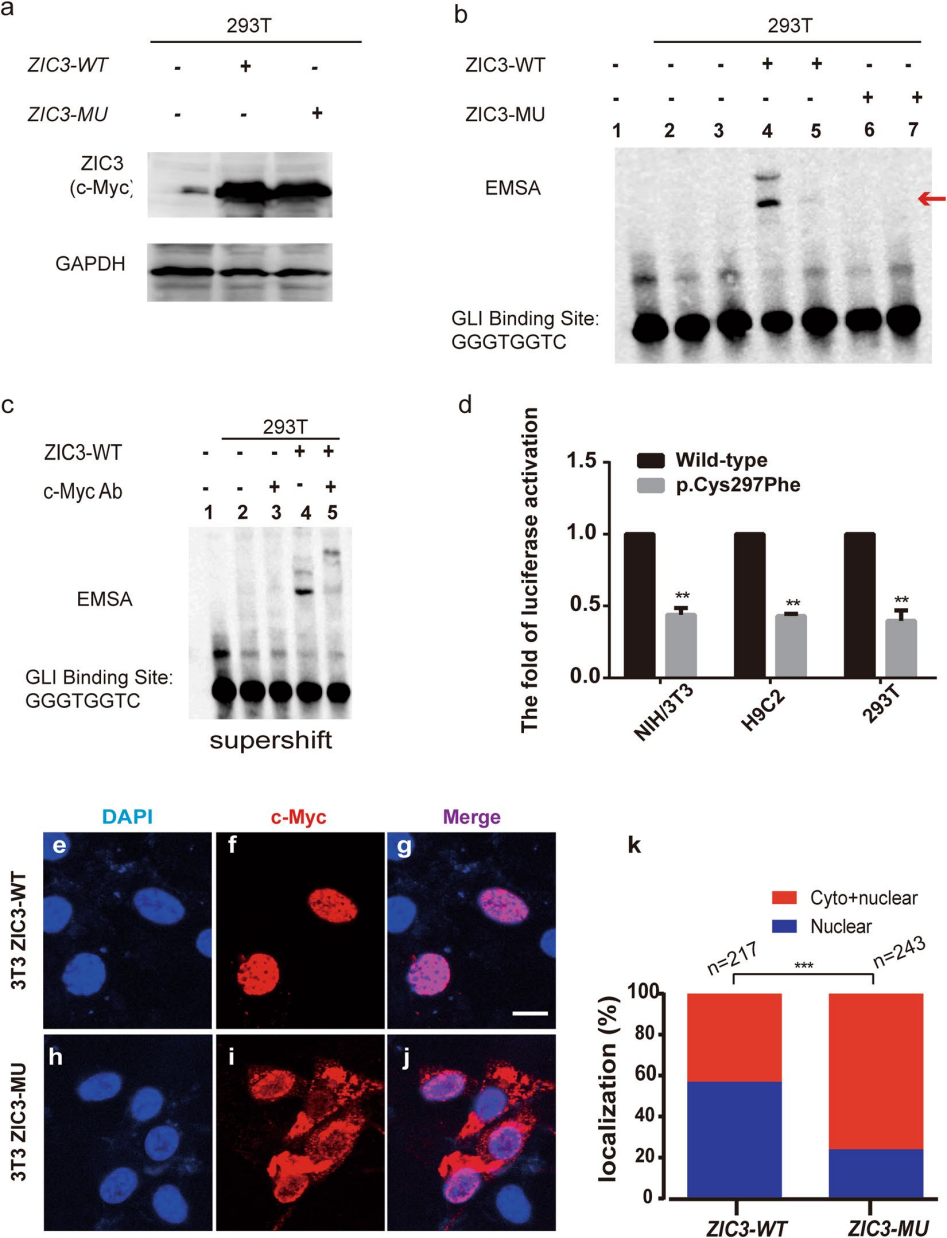
The *ZIC3* mutation causes functional changes in cell lines. (**a**) A western blot showed that the *ZIC3* wildtype (WT) and mutant (MU) forms are being expressed as equal levels for the EMSA experiment. (**b**) The ZIC3 domain binds to the GLI-binding site (GLBS). Line 1: Only the GLI probe without protein; line 2: GLI probe + protein in 293T cells; line 3: unlabeled competitor GLI probe + protein in 293T cells; line 4: GLI probe + protein in 293T cells transfected with p*ZIC3* (WT)-myc; line 5: unlabeled competitor GLI probe + protein in 293T cells transfected with p*ZIC3* (WT)-myc; line 6: GLI probe + protein in 293T cells transfected with p*ZIC3* (MU)-myc; line 7: unlabeled competitor GLI probe + protein in 293T cells transfected with p*ZIC3* (MU)-myc. The red arrow indicates the complex of GLIBS with the ZIC3 protein. (**c**) A supershift EMSA showed that c-Myc antibody could specific bind with whole cell lysate which transfected p*ZIC3*-myc construct. Line 1–4 added biotin-labeled probe. (**d**) The wild-type (p*ZIC3-myc*) or mutant (p.Cys297Phe) *ZIC3* construct was co-transfected into NIH/3T3, H9C2, and HEK-293T cells with pGL3-SV40 firefly and pRL-TK *Renilla* luciferase reporters. Luciferase activities were measured 24 hours post-transfection. The mean fold activation relative to the wild-type is shown. The results represent the average luciferase activation across a minimum of three individual experiments. Standard errors are indicated by vertical lines. “**” Denotes statistical significance (P < 0.05) by two-tailed, unpaired Student’s t-tests assuming unequal variance. (**e–j**) Subcellular localization of *ZIC3* determined by immunofluorescence in NIH3T3 cell lines. For each construct, anti-Myc (panels f,i) and DAPI (panels e,h) staining is shown individually and merged (panels g,j). The wild-type (WT) construct is located in the nucleus (panel f), but the C297A missense mutation construct is located in both the cytoplasm and nucleus (panel i). Scale bar indicates 63X magnification. (**k**) Percentage of localization. Cells transfected with the WT or MU *ZIC3* construct were classified as exhibiting either only nuclear localization or both nuclear and cytoplasmic localization. ***P < 0.0001 by the Chi-square test.

Previous studies have demonstrated that the ZIC3 protein binds to the GLIBS sequence (i.e., 5′-TGGGTGGTC-3′)^29,30^. To determine whether the *ZIC3* mutant (p.C297F) affects the GLIBS-binding ability of the ZIC3 protein, we performed electrophoretic mobility shift assays (EMSA) using wild-type ZIC3 protein and the mutant ZIC3 protein from transfected whole cell lysate, with a biotin-labeled probe. To confirm the *ZIC3* wildtype and mutant forms are being expressed, a western blot was perform showed in Fig. 3a.

As shown in Fig. 3b, in 293T cells, GLIBS bound to the wild-type ZIC3 protein (Fig. 3b, lines 4). However, this reaction was not detected for the mutant ZIC3 protein (Fig. 3b, line 6). To verify the binding specificity of the ZIC3 protein to the GLIBS, a mixture of the biotin-labeled GLIBS probe a 1X and the unlabeled GLIBS probe at 200X was used. When the unlabeled cold GLIBS probe was added to the reaction, binding was blocked (Fig. 3b, lines 3, 5, and 7). This EMSA result showed that the mutant ZIC3 (p.C297F) protein lacks the ability to bind GLIBS. Furthermore, a supershift control reaction was performed to verify that the band is actually caused by *ZIC3* (Fig. 3c).

We also performed a luciferase assay to determine the effect of the *ZIC3* mutation on gene transactivation. The mutant *ZIC3* construct was generated from the wild-type *ZIC3* plasmid via site-directed mutagenesis and contained a C-terminal Myc-tag. The wild-type and mutant *ZIC3* constructs were subsequently co-transfected into the NIH/3T3, H9C2, and 293T cell lines with an SV40 promoter-driven firefly luciferase reporter^22,31^. Our results showed that the *ZIC3* mutation in the C2H2 domain significantly decreases reporter gene transactivation compared with the wild-type controls (Fig. 3d).

### The *ZIC3* mutation influences the subcellular localization of the protein

As a transcription regulatory factor, the ZIC3 protein normally functions in the nucleus. To determine whether the mutant ZIC3 p.C297F protein changes the distribution of the protein in cells, we performed immunofluorescence analysis to detect its subcellular localization. The NIH/3T3 cell line was transiently transfected with the myc-tagged *ZIC3* construct used in the luciferase transactivation analysis. We scored and counted the cells exhibiting positive ZIC3 staining as showing nuclear, cytoplasmic, or both nuclear and cytoplasmic localization, as described in the Methods.

As shown in Fig. 3e–g, the wild-type ZIC3 protein was almost always located in the nucleus; however, the mutant ZIC3 protein could not enter the nucleus and accumulated at the nuclear periphery (Fig. 3h–j).

Based on cell counting, we observed that in cells transfected with the wild-type *ZIC3* construct, 50–55% of the cells exhibited protein localization in the nucleus, and 45–50% of cells exhibited both nuclear and cytoplasmic staining. In contrast, in the cells the transfected with the mutant (p.C297F) *ZIC3* construct, a large proportion (70–80%) of the cells displayed both nuclear and cytoplasmic protein localization, and a smaller percentage (20–30%) showed only nuclear localization (Fig. 3k).

These results suggest that the amino acid change in the zinc-finger domain of the *ZIC3* gene affects the normal function of the protein by blocking its entry into the nucleus.

### The *zic3* mutation-associated phenotype in zebrafish

**Figure 4:**
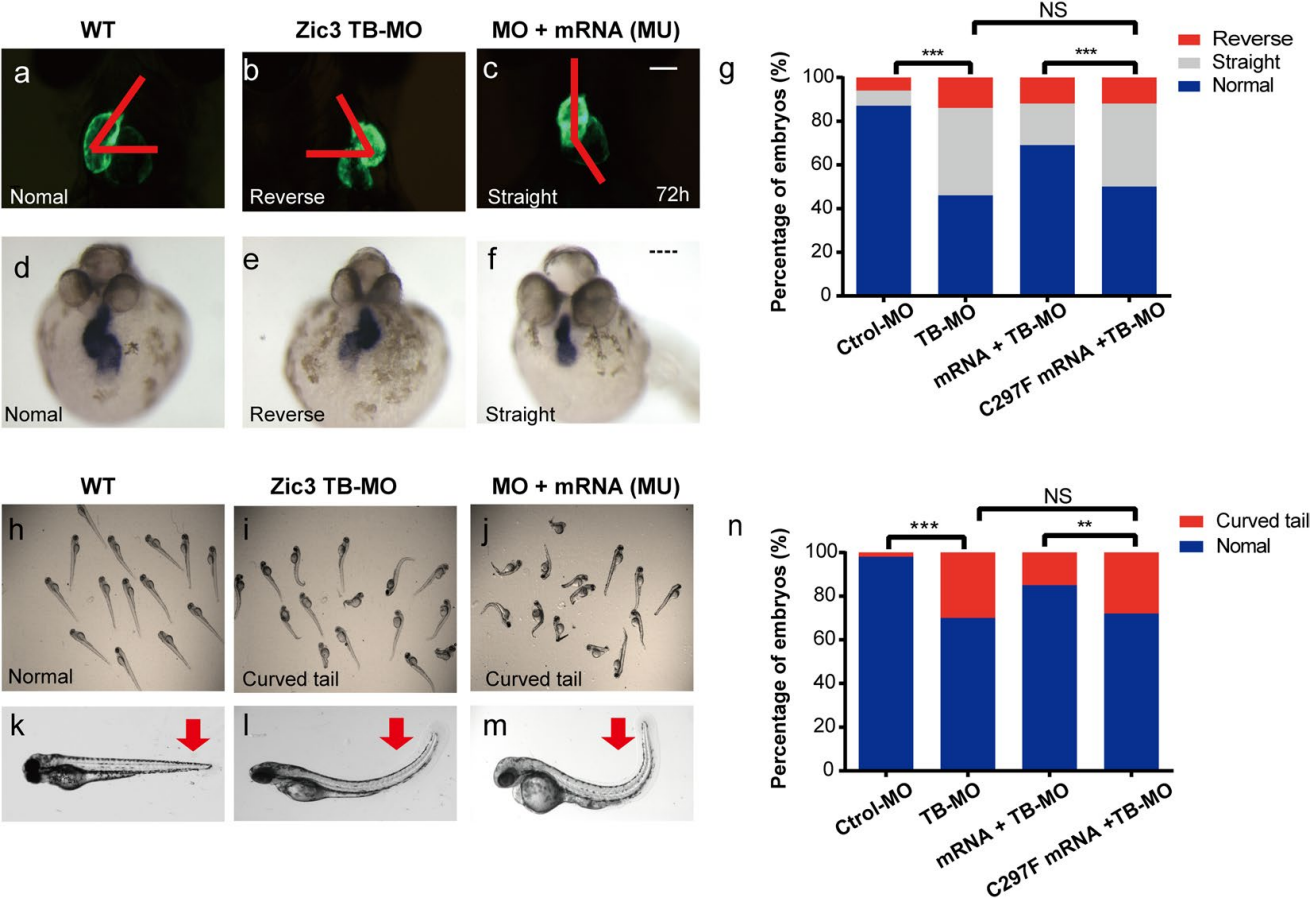
*zic3* knockdown in zebrafish embryos results in laterality defects. (**a**–**c**) Wild-type (WT) zebrafish showed normal cardiac looping, while in zebrafish injected with 2.0 ng of the *ZIC3* mRNA transcript blocking morpholino (TB-MO) or co-injected 2.0 ng of the TB-MO and 200 pg of mutant mRNA (Mu), the hearts were located at the midline or were reversed. The red line denotes the heart looping angle in normal, midline and reversed hearts. (**d**–**f**) *In situ* hybridization using a heart-specific probe (*cmcl2*) showed that WT embryos predominantly exhibited normal heart looping. In contrast, heart looping was often observed at the midline or reversed in *zic3* TB-MO-injected or mutant rescued embryos. (**g**) The graph shows the distribution of *cmcl2* expression observed in the embryos injected with the control MO (n = 168), 2.0 ng *zic3* TB-MO (n = 192), 100 pg *ZIC3* (WT) mRNA + 2.0 ng *zic3* TB-MO (n = 166), or 100 pg *ZIC3* (mu) mRNA + 2.0 ng *zic3* TB-MO (n = 232). The WT but not mutant human zic3 mRNA partially rescued the zebrafish heart looping defects. (**h**–**m**) In contrast to the WT zebrafish, curly tail was found in the *zic3* knockdown zebrafish injected with 2.0 ng of *zic3* TB-MO. Furthermore, the phenotypes of the mutant rescued group were extremely obvious. The red arrow indicates the curly tail. (**n**) The graph shows the distribution of the curly tail phenotype in the Control (Ctrol) MO (n = 254), 2.0 ng *zic3* TB-MO (n = 266), 100 pg *ZIC3* (WT) mRNA + 2.0 ng *zic3* TB-MO (n = 242), and 100 pg *ZIC3* (mu) mRNA + 2.0 ng *zic3* TB-MO (n = 261) groups. **P < 0.05, ***P < 0.005, NS indicates not significant by Fisher’s exact test. Scale bars: 16X (panel c) and 150 µM (panel f).

To assess any potential phenotype associated with the *zic3* mutation in zebrafish, we used antisense morpholino oligonucleotides (MO) to knockdown Zic3 protein expression in zebrafish. Embryos injected with the MO designed to block *zic3* mRNA translation (*zic3* TB-MO) exhibited morphological abnormalities including heart laterality and a curved body axis (Fig. 4). Other abnormalities, such as hydrocephalus or gross eye defects, were not observed in *zic3* knockdown zebrafish.

We next conducted rescue experiments through co-injection of human wild-type (WT) *ZIC3* mRNA or *ZIC3* mutant (c.890G > T) mRNA with *zic3* TB-MO separately to confirm that the defects observed in zebrafish embryos were specifically due to mutant *zic3*. In addition, we performed RNA *in situ* hybridization staining of the heart-specific marker *cmcl2* to confirm the of heart laterality defect phenotype.

Zebrafish co-injected with WT *ZIC3* mRNA and *zic3* TB-MO exhibited partial rescue of the observed heart looping defects (the percentage of normal embryos improved from 46% to 69%, P = 0.0022; Fig. 4a–g) and curved tail defects (the percentage of normal embryos increased from 70% to 85%, P = 0.0171; Fig. 4h–n). In contrast, the co-injection of zebrafish with mutant *zic3* mRNA failed to rescue either the heart looping defects (the percentage of normal hearts was 46% *versus* 50%, P = 0.8306; Fig. 4a–g) or the ventralized phenotype (the percentage of the curved tail phenotype was 70% *versus* 77%, P = 0.3364; Fig. 4h–n). Our results obtained using three independent experiments of MO injections suggest that *zic3* is required for normal heart laterality and a normal LR body axis in zebrafish. For each injection experiment, a minimum of 100 embryos were analyzed. These results suggest that this novel variant of *ZIC3* is both pathogenic and involved in HTX.

## Discussion

The establishment of left-right axis development is critical for normal organogenesis and provides the basis for correct heart looping^5^. However, the exact mechanisms that establish this asymmetry and drive heart development and differentiation are still largely unknown. Many genes associated with LR asymmetry disorders have recently been identified^32^. We consider gene panels identified through NGS to be more clinically applicable than those obtained through exome sequencing because of faster turnaround times, higher and more reliable coverage, and the ability to avoid incidental findings. In the current study, we developed a customized panel for testing variations in 22 genes related to LR asymmetry and cilia function in an attempt to identify variants responsible for heterotaxy. In 33% of our cases, 21 missense mutations with likely functional effects were predicted in seven genes: *DNAH5*, *ARMC4*, *MEGF8*, *SHROOM3*, *NPHP4*, *ACVR2B* and *ZIC3*. Thirteen of these mutations were low-MAF SNPs, and eight were found for the first time in our study. In particular, one hemizygous mutation c.890G > A (p.C297F) in the *ZIC3* gene was identified in the present study.

The *ZIC3* gene, a member of the ZIC family gene, plays a critical role not only in the maintenance of stem cell pluripotency but also in the development of LR asymmetry. Moreover, *ZIC3* is considered to act as a transcription factor, due to its ability to bind DNA and activate transcription^33^. Since the first gene was found to be associated with X-linked HTX through linkage analysis in five families in 1997, more than 36 pathogenic variants of the *ZIC3* gene have been identified, providing substantial evidence that either loss or aberrant function of the ZIC3 protein is among the causes of heterotaxy^8,10–12,22,31,34^.

The ZIC3 protein consists of five Cys2His2 (C2H2)-type zinc fingers. The second C2H2 zinc-finger domain (ZF2), from amino acid 295 to 322 in the ZIC3 protein, includes part of a nuclear localization signal (NLS) and nuclear export signal (NES) region^35,36^. Variants located in or around this region have been shown to result in functional defects that disrupt an NLS/NES. A previous study by Ware *et al*. showed that mutations p.H286R, p.Q292X and p.T323M mutations altered the subcellular localization from nuclear to cytoplasmic and caused aberrant reporter gene transactivation^37^. Moreover, Cowan *et al*. showed that p.Glu291GlyfsX53 and p.His318Asn in the *ZIC3* gene resulted in the same loss of function *in vitro*^22^. In the current study, the identified missense variant p.C297F was located in the ZF2 domain-combined NLS/NES region. This amino acid is highly conserved across several species. Additionally, this mutation was absent from the 1000 Genome and ExAc databases and was predicted it to be disease-causing by all predictive *in silico* programs. Consistent with previous research, this mutation decreased transactivation and altered the subcellular localization in 3T3 cell lines. Moreover, EI Malti *et al*. found a hemizygous variant at the same amino acid of *ZIC3* (c.889T > G, p.C297G) in a HTX fetus who was subject of a termination of pregnancy, which means that Cysteine is critical for ZIC3 function^38^. These findings suggest that the ZF2 domain and the NLS/NES region are essential for ZIC3 function in living organisms.

In addition to aberrant reporter gene transactivation and abnormalities in subcellular localization, this mutation (p.C297F) was found to affect protein structure and DNA-binding ability. Our protein structure prediction model using SWISS-MODEL software demonstrated that one ligand was lost in the mutant ZIC3 protein. Given that ZIC3 is known to physically and functionally interact with GLI proteins and to bind to the GLIBS sequence (5′-TGGGTGGTC-3′), which is critical for the Hedgehog signaling pathway, EMSA was performed to test the protein-DNA-binding reaction of mutant ZIC3 with GLIBS^29^. We noted obvious interactions between wild-type ZIC3 and GLIBS, whereas the mutant (p.C297F) ZIC3 protein showed no interaction with GLIBS. This result is consistent with a previous study demonstrating that ZF domains were necessary for the binding of ZIC3 to GLIBS^29^. Mutations in the ZF2 domain may cause the loss of DNA-binding ability and subsequent alterations in gene expression, which may lead to reduced Hedgehog signaling, ultimately resulting in HTX or congenital heart disease.

To combine our genetic findings with clinical phenotypes, we performed *in vivo* experiments using a zebrafish model, which is an attractive model for studying the function of genetic variations in cardiovascular disease^39^. Zic3 expression in zebrafish is restricted to the dorsal half of the blastoderm at early stages, before laterality develops^40^. A study in ZIC3 mutants overexpressing either wild-type *ZIC3* mRNA or mutant *ZIC3* mRNA showed that overexpression of wild-type *ZIC3* mRNA resulted in cardiac laterality defects, posterior truncation or embryo lethality. However, the examined mutants (p.Val288Serfs*50, p.His281Argfs*62, p.Asn371His) exhibited less extensive phenotypes, indicating a partial loss of function^31^. To confirm the role of *Zic3* in establishing LR asymmetry, we used an antisense MO to knockdown the expression of zebrafish *zic3*. Depletion of *zic3* in zebrafish resulted in abnormal heart looping and curved tail phenotypes, similar to the heart malposition observed in HTX patients. Co-injection of the *zic3* TB-MO and human wild-type *ZIC3* mRNA significantly improved the phenotypic spectrum resulting from *zic3* depletion. In contrast, co-injection of the *zic3* TB-MO and human mutant (p.C297F) *ZIC3* mRNA failed to rescue the observed laterality defects, suggesting that these variants are indeed pathogenic. In accordance with a study from Cast AE *et al*., LR patterning is disrupted in *zic3* morphant zebrafish^41^.

Collectively, the analyses conducted in the present study allowed us to assess the functional consequences of a novel *ZIC3* variant (c.890G > T) identified in HTX patients in both cell lines and zebrafish models, demonstrating that the mutation resulted in loss of function. Both *in vitro* and *in vivo* analyses confirmed that this novel mutation in the *ZIC3* gene significantly altered ZIC3 protein function, suggesting that *ZIC3* is involved in the establishment of LR asymmetry. These results provide evidence for further research on the target genes that are regulated or transcriptionally activated by *ZIC3* to explore the underlying mechanisms whereby *ZIC3* affects LR axis development.

## Conclusion

In summary, using a customized Ampliseq panel strategy based on NGS, we identified 21 predicted mutations, among which one novel hemizygous mutation in the *ZIC3* gene correlated with X-linked heterotaxy is reported for the first time. Both *in vivo* and *in vitro* results provided powerful evidence of an association between the novel *ZIC3* c.890G > T variant and HTX and CHD. Furthermore, our study confirmed and extended previous observations regarding the function of the *ZIC3* gene in LR asymmetry.

## Methods

### Patient recruitment and DNA extraction

The collection of samples from patients was conducted in accordance with the Declaration of Helsinki and was approved by the Ethics Committees of Children’s Hospital of Fudan University (CHFU). Informed consent was obtained from the parents or guardians of the children.

Sixty-six cases diagnosed as CHD and heterotaxy and the corresponding trio families were recruited from the Cardiac Center of the hospital in Shanghai, China, between August 2012 and December 2015. Each participant was categorized as having situs inversus, heterotaxy, or isolated heterotaxy spectrum CHD based on the classification described by Ware^37^. Genomic DNA samples from the whole blood of all patients as well as their nuclear family wherever available were extracted using the QIAamp DNA Blood Mini Kit (Qiagen). The DNA concentration was determined using a NanoDrop spectrophotometer (ND-1,000, Thermo Fisher Scientific, Waltham, MA).

### HTX panel design, NGS sequencing and data analysis

In this study, a customized HTX gene panel multiplex enrichment kit was designed using the Ion AmpliSeq strategy (Life Technologies, USA) with baits for the following twenty-two genes: *NPHP4*, *LEFTY1*, *LEFTY2*, *CFC1*, *ACVR2B*, *TGFBR2*, *RPSA*, *CRELD1*, *SHROOM3*, *DNAH5*, *GJA1*, *FOXH1*, *INVS*, *ARMC4*, *NODAL*, *NET43*, *BCL9L*, *NEK8*, *CCDC11*, *MEGF8*, *SMAD2* and *ZIC3*. A total of 567 amplicons were designed (details in Supplemental Table 1). Primers covering all exons and at least 10 bp of all intron/splice sites of these genes were designed online using the software available at https://www.ampliseq.com/.

Following the protocol, libraries were constructed using the Ion AmpliSeq Library Kit v2.0 (Life Technologies, USA). The libraries were prepared using kits for fragmentation (Ion Shear, Life Technologies). Various samples were distinguished by different barcodes using adaptors and barcode ligation (Ion Xpress Barcode Adapters Kit, Life Technologies). The concentrations of each library were confirmed using a TaqMan Quantification Kit (Life Technologies). To clonally amplify pooled barcoded libraries, the Ion OneTouch 2 system with the Ion PGM Template OT2 200 Kit (Life Technologies, USA) was used. Ion sphere particles (ISP) were enriched using the E/S module protocol. The enriched template-positive ISPs were loaded and sequenced on an Ion 316™ Chip by PGM (Life Technologies, USA)^42^. The coverage of these genes was greater than 96.3%, with an average reads depth greater than 428X for each targeted nucleotide. Samples with low coverage (<5 reads for locus) were excluded from the study.

For each case, base calls were detected with Torrent Suite software, and raw sequencing data were aligned against the human reference genome GRCh37/hg19 (NCBI) using NextGENe software, which can also call variants with default alignment settings. Single-nucleotide variations (SNVs) were aligned based on the following criteria: (1) the variant was detected on both strands of the sequence reads; (2) the minimum coverage of reads was no less than 10×; (3) at a particular site, the variant reads represented more than 20% of the sequence reads; and (4) the targeted region covered all exons and at least 20 bp of all intron/splice sites^18^. The variants filtered from NextGENe software and confirmed by Sanger sequencing were compared with the dbSNP (http://www.ncbi.nlm.nih.gov/projects/SNP/), 1000 Genomes (http://www.1000genomes.org), and Exome Aggregation Consortium (ExAc) databases (http://exac.broadinstitute.org/) as well as our laboratory’s internal databases that contain data from more than 3500 non-cardiac disease cases. Additionally, the risk of SNVs was predicted using the silico tools SIFT (http://sift.jcvi.org/), PolyPhen2(http://genetics.bwh.harvard.edu/pph2/) and MutationTaster (http://www.mutationtaster.org/)^42–44^ and confirmed via Sanger sequencing. The primer sequences used to analyze each variant via polymerase chain reaction are listed in Supplementary Table S2.

### *ZIC3* amino acid sequence conservation and protein structure prediction

The conservation of all the amino acids altered by missense mutations was estimated by aligning genes from *Homo sapiens* with those of *Mus musculus*, *Danio rerio*, *Numida meleagris*, *Columba livia*, *Anas platyrhynchos*, and *Heterocephalus glaber* using NCBI Blast (https://blast.ncbi.nlm.nih.gov/Blast.cgi). Wild-type and mutant ZIC3 protein structures were predicted using SWISS-MODEL (https://swissmodel.expasy.org/?pid=smh01)^45^.

### *ZIC3* expression plasmid construct, mutagenesis analysis and luciferase assay

The wild-type *ZIC3* plasmid was constructed by subcloning the entire human *ZIC3* (NM_003413) open reading frame (ORF) into the pCMV6 expression vector with a C-terminal Myc-tag (Origene, RC220375). The mutant *ZIC3* construct was generated from the wild-type *ZIC3* sequence via the Fast Mutagenesis System (Transgen Biotech, China) and confirmed by Sanger sequencing. The primers used to generate the mutant *ZIC3* plasmid were as follows: mutant-F: 5′-AGAACAACCACGTCTTCTACTGGGAGGAGTG-3′ and mutant-R: 5′-AAGACGTGGTTGTTCTGCTCCGGGCCC-3′.

The 293T, NIH/3T3 and H9C2 cell lines were grown in high-glucose Dulbecco’s modified Eagle medium (Gibco) with 10% fetal bovine serum (Gibco) and 1× Pen/Strep (Thermo Fisher Scientific) at 37 °C under 5% CO_2_. Lipofectamine 3000 (Invitrogen) was used for transfection according to the manufacturer’s protocol. The wild-type or mutant p*ZIC3-myc* construct was co-transfected with an SV40 luciferase reporter plasmid (pGL3-SV40, Promega) and a *Renilla* luciferase reporter (pRL-TK, Promega). The pGL3 basic plasmid (Promega) was used to normalize the results. Cells were harvested 48 hours after transfection, and luciferase activity was determined using the Dual Luciferase Reported Assay System (Promega). Firefly luciferase activity was normalized to *Renilla* luciferase activity. The fold activation of the ZIC3 mutant was calculated with respect to the wild-type values. The results for each construct represent the average values obtained from a minimum of three independent experiments repeated in triplicate.

### Western blot

The 293T cells which were transfected with wild-type and mutant *ZIC3* plasmid were harvested at 48 hours after transfection. Then, we used RIPA lysis and extraction buffer (Thermo Scientific, USA) containing halt protease/phosphatase inhibitor cocktail (Thermo Scientific, USA) to effectively lyse and extract protein from cells on ice. The protein concentrations were determined using the BCA protein assay kit (Takara) according to the manufacturer’s instructions, with bovine serum albumin (BSA) as the standard. Then, 30 μg of protein was loaded onto and separated in 10% sodium dodecyl sulfonate-polyacrylamide gels, followed by an electrophoretic transfer onto a nitrocellulose membrane (Whatman, GE Healthcare Life Sciences, UK). After this, the membrane was blocked in phosphate-buffered saline (PBS) with 1% Tween-20 (PBST) containing 5% BSA for 1 hour at room temperature to prevent non-specific antibody binding. Appropriate primary antibodies were incubated with the membrane, followed by peroxidase-conjugated anti-rabbit antibody (dilution at 1:5,000; Pierce), and visualized by enhanced chemiluminescence (Pierce). The primary antibodies that were used were mouse monoclonal anti-Myc (ab32, Abcam, UK) and anti-GAPDH (ab9482, Abcam, UK) was used as a loading control.

### Electrophoretic mobility shift assay (EMSA)

293T cells were collected 48 hours after transfection with p*ZIC3-myc* or p*ZIC3* (Mu)-*myc*, and whole-cell extracts were obtained using RIPA lysis and extraction buffer (Thermo Scientific). The obtained protein concentration was measured with a BCA protein assay kit (Takara). Biotin-labeled double stranded oligonucleotide probes containing a GLI-binding site (GLIBS) were generated with the following primers:

F: 5′-GCATCTGTGATTTTCGTCTTGGGTGGTCTCCCTCCTGTAGGAATTCG-3′; R: 5′-CGAATTCCTACAGGAGGGAGACCACCCAAGACGAAAATCACAGATGC-3′.

The binding experiment was performed using a LightShift Chemiluminescent EMSA Kit (Thermo Scientific) according to the manufacturer’s protocols. One picomole of biotin-labeled probe was incubated with 10 μg of a whole-cell extracts from 293T cells or cells transfected with p*ZIC3-myc* or pZ*IC3* (*Mu*)*-myc* separately for 10 min in 10X binding buffer mixed with poly (dI·dC), 1% NP-40 and 50% glycerol. A 200-fold molar excess of unlabeled probe was added to the reaction for competition experiments. The reaction mixture was subsequently subjected to electrophoresis in a 6% polyacrylamide gel at 100 V for 30 min. The gel was then transferred to a positive nylon membrane at 384 mA for 50 min and detected using a stabilized streptavidin-horseradish peroxidase conjugate and photographed using a Fuji film Las3000 Luminescent Image Analyzer (Fuji Life Sciences, Tokyo, Japan).

To confirm the band was actually caused by ZIC3, a supershift control reaction was performed by adding 1ul of mouse monoclonal anti-Myc antibody (ab32, Abcam, UK) to the whole cell-extracts of 293T cells transfected with p*ZIC3-my*. The protocol was same as EMSA according to the manufacturer’s instruction.

### Immunocytochemistry and subcellular localization

NIH3T3 cells were seeded onto 20 mm-diameter glass-bottom cell culture dishes (Nest scientific, USA) at 0.6 × 10^5^ cells/ml one day before transfection with either the wild-type or mutant p*ZIC3-myc* construct. For each transfection, 2.5 μg of plasmid DNA was transfected using the Lipofectamine 3000 Transfection reagent (Invitrogen, USA). After 24 hours, the transfected cells were fixed in 4% paraformaldehyde/PBS for 20 min at room temperature and then washed in 0.5% TritonX-100/PBS (PBST) three times. Following an additional 20 min in PBST, the cells were blocked in 3% fetal bovine serum/0.2% TritonX-100/PBS for 1 hour at room temperature. The cells were subsequently incubated overnight at 4 °C in a 1:200 dilution of an anti-c-Myc mouse monoclonal antibody (ab32, Abcam, UK), washed three times in PBST, incubated for 2 hours in a 1:500 dilution of Alexa Fluor 594 goat anti-mouse IgG (ab150116, Abcam, UK) in the dark, and then washed two additional times in PBST. Nuclear staining was performed with 1:1000-diluted DAPI, followed by washing with PBST. The slides were finally imaged using a Leica TCS SP8 Laser Scanning Confocal Microscope.

### Zebrafish *zic3* knockdown and rescue experiments

*Danio rerio* of the Tu and transgenic cmcl2-eGFP strains were reared under standard aquaculture conditions at 28.5 °C with a 14/10 light/dark cycle. Embryos were obtained after natural group mating and cultured in egg water (17 mM NaCl, 0.20 mM KCl, 0.18 mM Ca (NO3)_2_, 0.1 mM MgSO4, 1.5 mM HEPES buffer at pH 7.1–7.3, with 0.6 µM methylene blue) with or without 1-phenyl-2-thiourea (PTU; Sigma) to prevent pigmentation. All zebrafish experiments were approved by the CHFU, and the methods of the zebrafish experiments were performed in accordance with the approved guidelines and regulations.

Antisense MOs were purchased from Gene Tools, LLC. To knockdown *zic3*, we designed one MO to bind to the start codon and block translation: *zic3* TB-MO 5′-GCGCTATCAAGGAGCATAGTCATT-3′. MO sequences were aligned with the *Danio rerio* genome using UCSC Blast and NCBI Blast to confirm the specificity for the *zic3* genomic region. Different amounts of each MO were injected to determine the optimum dose. Ultimately, we performed the injection with 2.0 ng of *zic3* TB-MO. A standard MO (5′-CCTCTTACCTCAGTTACAATTTATA-3′) purchased from Gene Tools, LLC was used as a control.

For the MO rescue experiments, the wild-type and mutant human *ZIC3* cDNAs were cloned from the p*ZIC3-myc* plasmid into the pCS2+ vector plasmid (Addgene, USA) using the In-Fusion® HD Cloning Kit (Takara Bio, USA) using the appropriate primers (Supplementary Table S2). The wild-type and mutant mRNAs were synthesized with the mMESSAGE mMACHINE SP6 ULTRA Kit (Ambion, Inc). Then, 100 pg of mRNA was co-injected with 2.0 ng of *zic3* TB-MO in 1-cell-stage embryos^31^. As a control, equal volumes of solution were injected. A minimum of three independent experiments were performed.

### Zebrafish phenotype analysis and whole-mount *in situ* hybridization (WISH)

All surviving embryos at 72 hours post-injection were collected from each group and carefully observed using a Leica M205C inverted microscope. The number of embryos with cardiac looping defects, curved tail, and edema phenotypes were counted. A minimum of three independent experiments were performed. A *cmcl2* DIG-labeled probe was used to confirm the phenotypes via WISH as previously described^46^.

### Statistical analysis

Unpaired Student’s t-tests were used to analyze the luciferase data. Statistical analysis for functional assessment was performed using the Chi-square test with GraphPad Prism 6.0 Software. Values were considered significantly different at p < 0.05.

### Data availability

The datasets generated during and/or analysed during the current study are available from the corresponding author on reasonable request.

## Electronic supplementary material

Dataset 1
