# Supplementary material for: A novel ZIC3 gene mutation identified in patients with heterotaxy and congenital heart disease
Source: Sci Rep. 2018 Aug 17;8:12386. doi: 10.1038/s41598-018-30204-3 (PMC6098004; doi:10.1038/s41598-018-30204-3)
Supplement: Supplementary file 1 — Dataset 1 [file 41598_2018_30204_MOESM1_ESM.doc]

**A novel *ZIC3* gene mutation identified in patients with heterotaxy and congenital heart disease**

Shuolin Li^1^, Sida Liu^1^, Weicheng Chen^1^, Yuan Yuan^1^, Ruoyi Gu^1^, Yangliu Song^1, 2^, Jian Li^1^, Yinyin Cao^1^, Yixiang Lin^1^, Jun Xu^1^, Huijun Wang^1,2^, Duan Ma^2, 3^, Xiaojing Ma^1, 2^, Wei Sheng^1, 2, *^, Guoying Huang^1, 2, *^

^1^Children’s Hospital of Fudan University, Shanghai, China, 201102

^2^Shanghai Key Laboratory of Birth Defects, Shanghai, China, 201102

^3^Key Laboratory of Molecular Medicine, Ministry of Education, Shanghai Medical College, Fudan University, China, 200000

^*^Corresponding author:

Guoying Huang, Children’s Hospital of Fudan University, 399 Wanyuan Road, Shanghai, China, 201102; Email: gyhuang@shmu.edu.cn

Wei Sheng, Shanghai Key Laboratory of Birth Defects, Shanghai, 399 Wanyuan Road, China, 201102; Email: Sheng4616@126.com

First Author:

Shuolin Li

399 Wanyuan Road, Shanghai, China, 201102

Email: [specialshuo@126.com](mailto:specialshuo@126.com)

Tel：+86 186-1673-0157

SUPPLEMENTARY Tables

**Supplementary Table S1. List of 22 candidate genes selected for targeted sequencing.**

^1^From the NCBI Gene database; assembly GRCh37/hg19

| Gene name | Abbreviation | Locus | Refseq Transcript^1^ | OMIM | Mean Function^2^ | Overal  Coverage (%) |
| --- | --- | --- | --- | --- | --- | --- |
| *Nephronophthisis 4* | NPHP4 | 1p36 | NM_015102 | 607215 | localized to actin- and microtubule-based structures  Signaling by hippo and wnt pathway | 99.9 |
| *Left-Right Determination Factor 1* | LEFTY1 | 1q42.1 | NM_020997 | 603037 | Heart morphogenesis  Signaling by NODAL, TGF-beta signaling pathway | 81.5 |
| *Left-Right Determination Factor 2* | LEFTY2 | 1q42.1 | NM_003240 | 601877 | Left-right asymmetry determination  SMAD protein signal transduction | 83.2 |
| *Cryptic Family 1* | CFC1 | 2q21.1 | NM_032545 | 605194 | Determination of left/right asymmetry  Signaling by NODAL | 95.6 |
| *Activin A Receptor Type 2B* | ACVR2B | 3p22 | NM_001106 | 602730 | Heart development  BMP signaling pathway | 95.9 |
| *Transforming Growth Factor Beta Receptor 2* | TGFBR2 | 3p22 | NM_003242 | 190182 | ATP binding, SMAD binding,  Notch signaling pathway, heart looping | 100 |
| *Ribosomal protein SA* | RPSA | 3p22.2 | NM_002295 | 150370 | Laminin binding  Cell adhesion, differentiation, migration, signaling, neurite outgrowth and metastasis | 96.4 |
| *Cysteine Rich with EGF Like Domains 1* | CRELD1 | 3p25.3 | NM_015513 | 607170 | Cardiac septum development  Calcium ion binding | 93.1 |
| *Shroom Family Member 3* | SHROOM3 | 4q21.1 | NM_020859 | 604570 | Actin filament binding  Cell morphogenesis | 92.9 |
| *Dynein Axonemal Heavy Chain 5* | DNAH5 | 5p15.2 | NM_001369 | 603335 | Microtubule moto activity  Cilium assembly, movement | 100 |
| *Gap Junction Protein Alpha 1* | GJA1(cx43) | 6q22.31 | NM_000165 | 121014 | Gap junction  Heart looping, development | 100 |
| *Fork Head Box H1* | FOXH1 | 8q24.3 | NM_003923 | 603621 | SMAD binding, gap junction protein  Aorta morphogenesis, heart looping | 70.3 |
| *Inversin* | INVS | 9q31 | NM_014425 | 243305 | Wnt signaling pathway  Left-right axis determination | 100 |
| *Armadillo Repeat Containing 4* | ARMC4 | 10p12.1 | NM_018076 | 615408 | Ciliary and flagellar movement  Heart development | 100 |
| *Nodal Growth Differentiation Factor* | NODAL | 10q22.1 | NM_018055 | 601265 | BMP signaling  Growth factor activity | 100 |
| *N-acetyltransferase 10* | NET43  (NAT10) | 11p13 | NM_024662 | 609221 | ATP binding  rRNA modification | 100 |
| *B-cell CLL/lymphoma 9-like* | BCL9L | 11q23.3 | NM_182557 | 609004 | Beta-catenin binding  Regulation of cell morphogenesis | 93.8 |
| *NIMA related kinase 8* | NEK8 | 17q11.1 | NM_178170 | 609799 | ATP binding  Heart development | 98.3 |
| *Cilia and Flagella Associated Protein 53* | CFAP53/  CCDC11 | 18q21.1 | NM_145020 | 614759 | Cilium assembly, movement | 100 |
| *multiple EGF like domains 8* | MEGF8/  CRPT2 | 19q12 | NM_001410 | 604267 | Calcium ion binding  BMP signaling pathway, Embryonic heart tube left/right pattern formation | 95.6 |
| *SMAD family member 2* | SMAD2 | 19q21.1 | NM_001003652 | 601366 | SMAD binding  NODAL signaling pathway | 100 |
| *Zic family member 3* | ZIC3 | Xq26.3 | NM_003413 | 300265 | Transcription factor  Heart looping | 100 |

^2^Gene function annotation is from Genecard website.

**Supplementary Table S2.** **Primers designed for variant confirmation**

| Primer name | CDS | Primer sequence | Amplicon size(bp) |
| --- | --- | --- | --- |
| DNAH5_S1F | c.13364C>A | cctttacagcagagtcagtgatg | 824 |
| DNAH5_S1R |  | gaaagcttggctccgaatc |  |
| DNAH5_S2F | c.12367C>T | tgccttcactctctctgtcatt | 805 |
| DNAH5_S2R |  | gctctgattctttcacactgcc |  |
| DNAH5_S3F | c.12595C>T | tcccctccccaaagtctctt | 698 |
| DNAH5_S3R |  | aacaagcttccccacccac |  |
| DNAH5_S4F | c.10169A>G | tcctcagactgaatacacctagg | 900 |
| DNAH5_S4R |  | ctaacaccagaagccacagg |  |
| DNAH5_S5F | c.7123A>T | cctcagcccctagaacagtg | 678 |
| DNAH5_S5R |  | cagggtttgaatgtcccagt |  |
| DNAH5_S6F | c.6053T>C | tgcgaaaatgttgccaaata | 840 |
| DNAH5_S6R |  | gctgctaaaatgagccagatg |  |
| DNAH5_S7F | c.12472C>T | ggaatttggggagggatctga | 728 |
| DNAH5_S7R |  | attctttcacactgccggga |  |
| DNAH5_S8F | c.9781A>G | atggtgggtgggcatttaca | 803 |
| DNAH5_S8R |  | tgaggacccaggactcttgg |  |
| DNAH5_S9F | c.9236G>A | tcccttcagatttcctccagc | 982 |
| DNAH5_S9R |  | tctttgattctcagcattgcagc |  |
| ARMC4_SF | c. 1679C>T | atgccagatgtgtgtaacctc | 672 |
| ARMC4_SR |  | gcagcaggtctcactatctct |  |
| MEGF8_S1F | c.3109C>T | ctcctctctccctgtcattgtt | 600 |
| MEGF8_S1R |  | aacctctttcaactaggtcctcc |  |
| MEGF8_S2F | c.8068C>A | ctcaagtcgagccgcttcta | 700 |
| MEGF8_S2R |  | ggttgtcctggctcaacagt |  |
| SHROOM3_S1F | c.580C>A | agaccaagaaagaccaaggca | 824 |
| SHROOM3_S1R |  | agcacacttaagcactgaagga |  |
| SHROOM3_S2F | c.4726A>G | gacaacctcctgaccagctg | 803 |
| SHROOM3_S2R |  | agcaagttcacatctcgggg |  |
| SHROOM3_S3F | c.2905C>T | aaacggggagctgaagttgg | 594 |
| SHROOM3_S3R |  | atcccatttctctgcgggc |  |
| NPHP4_S1F | c.2198G>A | aactccctagccagcctcat | 846 |
| NPHP4_S1R |  | cccagtcattcgcgtatcca |  |
| NPHP4_S2F | c.880G>A | gctgagaggatcatgctgtg | 599 |
| NPHP4_S2R |  | tccaaacttccaactccaaag |  |
| NPHP4_S3F | c.694C>T | tggggtagattttggactgc | 593 |
| NPHP4_S3R |  | aggggtccctaaagatccaa |  |
| NPHP4_S4F | c.3160C>T | ggtcgccgagttctttgagt | 831 |
| NPHP4_S4R |  | tcggaagggaccaaacaaca |  |
| ACVR2B_SF | c.1219G>A | atgtggtgaatcgaggtttgc | 821 |
| ACVR2B_SR |  | gacaccaaggcccctactag |  |
| ZIC3_S1F | c.890G>T | accgcccagtggccagcccg | 630 |
| ZIC3_S1R |  | cctgcgcaaacacatgaagg |  |
| ZIC3-InfusionF |  | tgttctttttgcaggatcccatcgat-  gccatgacgatgctcctggacggaggccc |  |
| ZIC3-InfusionR |  | acgactcactatagttctagatcagacg-  taccattcgttaaaattaggaggaagtccaggg |  |

All primers are listed in 5’🡺3’ direction
